# Resolvins Revisited: Methodological and Translational Gaps. Comment on Ghemiș et al. The Involvement of Resolvins in Pathological Mechanisms of Periodontal Disease Associated with Type 2 Diabetes: A Narrative Review. *Int. J. Mol. Sci.* 2024, *25*, 12784

**DOI:** 10.3390/ijms26178116

**Published:** 2025-08-22

**Authors:** Maxim A. Kriventsov, Olga A. Neprelyuk

**Affiliations:** 1 Pathomorphology Department, Medical Institute Named After SI Georgievsky, VI Vernadsky Crimean Federal University, Simferopol 295000, Russia; 2 Orthopedic Dentistry Department, Medical Institute Named After SI Georgievsky, VI Vernadsky Crimean Federal University, Simferopol 295000, Russia; oneprelyuk@mail.ru

The recent narrative review, titled “The Involvement of Resolvins in Pathological Mechanisms of Periodontal Disease Associated with Type 2 Diabetes: A Narrative Review” [1], addresses a critically important topic. Periodontitis and type 2 diabetes mellitus (T2DM) are chronic inflammatory conditions that exacerbate one another through shared immunometabolic pathways. The review focuses on the anti-inflammatory and pro-resolving lipid mediators—particularly specialized pro-resolving mediators (SPMs) such as resolvins—as emerging therapeutic targets. Given the central role of unresolved inflammation in both local (gingival) and systemic (metabolic) pathology, the investigation of SPMs is both timely and of high translational value.

Despite the relevance of the topic, the review lacks methodological transparency. No details are provided regarding how the literature search was conducted, the number of studies screened, excluded, or included, the types of studies assessed (e.g., in vitro, animal, or clinical trials), or whether systematic criteria were applied. Without such methodological rigor, the review’s findings are vulnerable to selection bias and cannot reliably support broad conclusions. Adherence to minimum reporting standards—such as those outlined by PRISMA for narrative or scoping reviews—would have significantly enhanced the reliability and reproducibility of the evidence synthesis [2].

Failure to follow the above-mentioned standard of data presentation may lead to uncritical presentation of the existing preclinical or clinical evidence, as well as some omissions in data. One such critical omission is the not mentioning of T-series resolvins (RvTs), also known as 13-series resolvins, which are biosynthesized from n-3 docosapentaenoic acid. While there are currently no preclinical or clinical studies directly investigating the effects of T-series resolvins (RvTs) on periodontitis, several preclinical studies provide strong indirect support for their potential relevance to periodontal disease due to their potent immunoresolving properties. First described by Dalli et al. in 2015 [3], RvT1–RvT4 have demonstrated potent activity in clearing neutrophil extracellular traps, enhancing macrophage efferocytosis and improving outcomes in infection and inflammation models [4]. Notably, RvT4 has also been shown to activate cholesterol efflux in lipid-laden macrophages via an SR-BI-dependent pathway, a mechanism with particular relevance to diabetes mellitus, where impaired macrophage lipid handling contributes to chronic inflammation and atherogenesis [5]. Their omission from the review narrows the scope of resolvin biology and excludes a key family with possible relevance to periodontal and systemic inflammatory modulation.

Beyond this, the review further adopts an overly optimistic stance toward resolvin families, not addressing existing disagreements related to omega-3 fatty acids, the primary source of resolvin biosynthesis. While preclinical studies consistently highlight the anti-inflammatory potential of omega-3-derived SPMs, the clinical landscape is far more complex. Numerous randomized controlled trials and meta-analyses have reported inconsistent, modest, or null effects of omega-3 supplementation on periodontal parameters, both in diabetic and non-diabetic populations [6,7]. According to the recent systematic reviews and meta-analyses, most of the currently available clinical studies with omega-3 fatty acids supplementation in patients with periodontitis are characterized by a high risk of bias and relatively low level of quality [8,9]. Not acknowledging these existing gaps in our state-of-the-art understanding of the metabolism of fatty acids, the review misses an opportunity to critically evaluate the translational challenges facing resolvin-based therapeutic strategies.

One underexplored but likely explanation for these clinical inconsistencies lies in interindividual variability in endogenous SPM biosynthesis. While many animal models benefit from controlled dietary and genetic backgrounds, human populations exhibit significant heterogeneity in omega-3 polyunsaturated fatty acids metabolism, enzyme activity (e.g., 15-LOX, COX-2), gut microbiota composition, and underlying inflammatory status. For instance, in healthy adults subjected to an endotoxin challenge combined with omega-3 intake, a temporal and individually variable rise in SPMs such as RvE1 and RvD1 was observed, indicating personalized metabolic responses [10]. Additionally, variations in microbiome composition and host genes (e.g., CYP enzyme polymorphisms) have been shown to affect SPM production, highlighting the role of host–microbe interactions in modulating individual SPM-generating capacity [11]. Furthermore, in patients with chronic inflammatory conditions—such as rheumatoid arthritis or metabolic syndrome—SPM biosynthesis may be impaired or dysregulated, thereby limiting the effectiveness of omega-3 supplementation. This has been demonstrated in diseases such as arthritis [12] and Alzheimer’s disease [13], where defective SPM synthesis or signaling appears to contribute to unresolved inflammation despite intake of omega-3 polyunsaturated fatty acids. Given these observations, we suggest a renewed focus on clinical studies that directly measure SPM levels—including resolvins, maresins, and lipoxins—before and after omega-3 polyunsaturated fatty acids supplementation in various inflammatory disorders. Correlating these biomarker changes with clinical outcomes could provide a more precise understanding of responders versus non-responders and support the development of personalized nutritional or pharmacological strategies.

As the authors rightly note, large-scale human clinical trials are needed to determine the optimal dosages, delivery methods, and long-term safety of resolvins. This is a critical point. In our view, two complementary translational strategies emerge. The first, as outlined in the review, involves the development of chemically stable formulations and targeted delivery systems—particularly those that enhance tissue-specific bioavailability, such as gingival-targeted gels for periodontitis or systemic analogs for diabetes-related conditions. The second, and perhaps more foundational, is to address the biological heterogeneity in natural SPMs biosynthesis. This would involve deepening our understanding of how omega-3 polyunsaturated fatty acids are enzymatically converted to resolvins in vivo. Bridging this mechanistic gap may ultimately enable more personalized or nutrigenomic approaches to SPMs modulation, reducing reliance on synthetic analogs and better predicting individual therapeutic response.

For the field to move forward, future studies should adopt standardized clinical trial designs with biomarker-driven endpoints and well-defined inclusion criteria. In addition, further research should explore the role of T-series resolvins in both periodontal and systemic inflammation, examine personalized predictors of SPM responsiveness (e.g., microbiome profiles, genetic variants), and continue to develop targeted delivery systems and stable analogs. Only through such an integrated and mechanistically informed approach can the full therapeutic potential of SPMs be realized in managing chronic inflammatory diseases such as periodontitis and type 2 diabetes.
